# The role of deficient pain modulatory systems in the development of persistent post-traumatic headaches following mild traumatic brain injury: an exploratory longitudinal study

**DOI:** 10.1186/s10194-020-01207-1

**Published:** 2020-12-03

**Authors:** Kelly M. Naugle, Christopher Carey, Eric Evans, Jonathan Saxe, Ryan Overman, Fletcher A. White

**Affiliations:** 1 Department of Kinesiology, School of Health and Human Sciences, Indiana University Purdue University Indianapolis (IUPUI), 901 West New York St., Indianapolis, IN 46202 USA; 2 Department of Health Sciences, School of Health and Human Sciences, IUPUI, 250 University Boulevard, Indianapolis, IN 46202 USA; 3 Trauma Department, Ascension St. Vincent Indianapolis Hospital, 2001 W 86th St, Indianapolis, IN 46260 USA; 4 Department of Neurology, Indiana University School of Medicine, GH 4700 Neur, IN 46202 Indianapolis, USA; 5 Department of Anesthesia, School of Medicine, Indiana University, 320 West 15th Street, Indianapolis, IN 46202 USA; 6 Stark Neuroscience Research Institute, School of Medicine, Indiana University, 320 West 15th Street, Indianapolis, IN 46202 USA

**Keywords:** Mild traumatic brain injury, Pain modulation, Conditioned pain modulation, Pain catastrophizing, Posttraumatic headache

## Abstract

**Background:**

Post-traumatic headache (PTH) is one of the most common and long-lasting symptoms following mild traumatic brain injury (TBI). However, the pathological mechanisms underlying the development of persistent PTH remain poorly understood. The primary purpose of this prospective pilot study was to evaluate whether early pain modulatory profiles (sensitization and endogenous pain inhibitory capacity) and psychological factors after mild TBI predict the development of persistent PTH in mild TBI patients.

**Methods:**

Adult mild TBI patients recruited from Level I Emergency Department Trauma Centers completed study sessions at 1–2 weeks, 1-month, and 4-months post mild TBI. Participants completed the following outcome measures during each session: conditioned pain modulation to measure endogenous pain inhibitory capacity, temporal summation of pain and pressure pain thresholds of the head to measure sensitization of the head, Pain Catastrophizing Scale, Center for Epidemiological Studies – Depression Scale, and a standardized headache survey. Participants were classified into persistent PTH (PPTH) and no-PPTH groups based on the 4-month data.

**Results:**

The results revealed that mild TBI patients developing persistent PTH exhibited significantly diminished pain inhibitory capacity, and greater depression and pain catastrophizing following injury compared to those who do not develop persistent PTH. Furthermore, logistic regression indicated that headache pain intensity at 1–2 weeks and pain inhibitory capacity on the conditioned pain modulation test at 1–2 weeks predicted persistent PTH classification at 4 months post injury.

**Conclusions:**

Overall, the results suggested that persistent PTH is characterized by dysfunctional alterations in endogenous pain modulatory function and psychological processes in the early stages following mild TBI, which likely exacerbate risk for the maintenance of PTH.

## Background

Approximately 1.7 million traumatic brain injuries (TBIs) occur in adults each year in the United States, with mild TBI accounting for the majority (~ 79%) of these injuries [1]. Posttraumatic headache (PTH) is one of the most common and persistent symptoms following a mild TBI, often disrupting recovery in civilian and military populations [2]. The International Headache Society defines posttraumatic headache as a secondary headache that develops within 7 days after the head trauma and is considered persistent when the headache lasts more than 3 months [3]. In civilian adult populations, prevalence rates of persistent PTH range from 30 to 90% following mild TBI, with 50–60% lasting at least 1 year [4–6]. In the military setting, a cross-sectional study showed that 98% of soldiers with mild TBI reported headaches, with 37% of those meeting diagnosis of PTH [7, 8]. Despite the high prevalence of PTH following mild TBI, the pathological mechanisms underlying the development of persistent PTH remain poorly understood.

Pain is modulated by complex endogenous systems that both facilitate and inhibit pain. Many chronic pain conditions are characterized by abnormal and unbalanced endogenous pain modulatory function (i.e., deficient descending pain inhibition and enhanced pain facilitation) [9–11]. Importantly, recent research indicates that mild TBI may exert deleterious effects on endogenous pain modulatory function, potentially underlying the elevated risk for intense and chronic PTH’s following injury. For example, Sahbaie and colleagues demonstrated enhanced sensitization to noxious mechanical stimuli in mice for up to 2 weeks following mild TBI [12]. Furthermore, mild TBI significantly diminished descending inhibition of pain in mice as measured by diffuse noxious inhibitory controls compared to a sham condition. Similarly, in a cross-sectional human study, our lab revealed decreased pressure pain thresholds of the head and decreased pain inhibition on the conditioned pain modulation (CPM) test in mild TBI patients within 2 weeks of injury compared to a non-mild TBI control group [13]. These results are in line with another study showing less efficient CPM in mild TBI patients within 72 h of the injury [14]. Furthermore, a cross-sectional human study demonstrated that mild TBI patients with chronic PTH (> 1 year post injury) exhibit diminished pain inhibitory capacity compared to control groups [15], with the magnitude of headache pain intensity correlating negatively with magnitude of pain inhibition. However, no prospective human studies have evaluated whether pain modulatory profiles in the early stages following mild TBI can predict the development of persistent PTH.

The purpose of this prospective pilot study was twofold. First, we sought to determine differences in pain sensitization and pain inhibitory capacity on quantitative sensory tests in mild TBI patients who develop persistent PTH compared to mild TBI patients who do not develop persistent PTH across 4 months post injury. Additionally, we evaluated differences in psychological factors across time, with a focus on depression and pain catastrophizing. Both depression and pain catastrophizing are elevated following mild TBI [14, 16] and have been associated with PTH [17, 18]; however, limited prospective research has evaluated their contribution to persistent PTH. Thus, headaches, psychological factors (i.e., depression, pain catastrophizing), sensitization of the head/neck area, and descending pain inhibition via dynamic quantitative sensory tests were measured in a sample of mild TBI patients within 2 weeks of injury, 1-month post injury, and 4-months post injury. We hypothesized that pain inhibition on the CPM test would be deficient and sensitization, pain catastrophizing, and depression would be elevated in those developing persistent headaches compared to those who do not. Secondly, we evaluated whether early pain modulatory (sensitization and endogenous pain inhibitory capacity) profiles and psychological factors after mild TBI predicted the development of persistent PTH’s in mild TBI patients. We hypothesized that sensitization of the head, deficient pain inhibitory capacity, and increased pain catastrophizing at 1–2 weeks post injury would predict the development of persistent posttraumatic headaches following mild TBI at 4 months post injury.

## Methods

### Participants

Fifty-five participants with mild TBI were enrolled in this study. Eleven participants dropped out of this study before completing the last study visit at 4 months. These 11 participants were no-shows for the last follow-up visit and/or did not return calls and emails of research staff to schedule the follow-up visit. Thus, 44 participants completed the study and will be the focus herein. Mild TBI participants had to have a mild TBI diagnosis according to the criteria recommended by the World Health Organization Task Force [19]: 1) a Glasgow Coma Scale score between 13 and 15 when examined at the emergency center, 2) no abnormal findings on a computed tomography scan of the brain to exclude secondary disorders such as hematoma, cerebral vein thrombosis, cerebral hemorrhage, or epilepsy, and 3) the presence of one or more of the following: confusion or disorientation, post-traumatic amnesia for less than 24 h, or a loss of consciousness for less than 30 min. The mild TBI could not be due to drugs, alcohol, medications, caused by other injuries or treatments for other injuries, caused by other problems (i.e. coexisting medical conditions, psychological trauma), or a penetrating craniocerebral injury. Exclusion criteria included: 1) chronic cardiovascular disease or uncontrolled hypertension, 2) metabolic disease, 3) neurological disease, 4) serious psychiatric conditions or hospitalization within the preceding year for psychiatric illness, 5) chronic headaches before the head injury, 6) current involvement in litigation, 7) chronic use of narcotics, and 8) fracture or polytrauma at the time of head injury. A subset of these participants were included in the data analysis of Carey et al., which compared mild TBI participants 1–2 weeks post injury to matched control participants [13].

#### Recruitment

The mild TBI participants were recruited from Level 1 trauma centers within hospitals located in the Indianapolis area. Potentially eligible patients had their electronic medical records screened by study recruiters to identify patients that met the inclusion/exclusion criteria. A mild TBI diagnosis was also confirmed by the attending Emergency Department (ED) Physician. Potentially eligible patients were then approached in the Emergency Room and handed a study information sheet describing important details of the study. If the patient expressed interest in the research, his/her identification was put into a secure database. At this point, the research staff would contact the potential participant within 48 h by phone or email to review the inclusion and exclusion criteria again with the participant as well as give particular details of the study to the potential participant. For those still interested, the laboratory session was scheduled within 2 weeks of the injury. Approximately, 33% of patients that were entered into the database by the recruitment staff were enrolled in this study. Patients in the database that were not enrolled 1) did not meet inclusion/exclusion criteria and therefore were not scheduled for first study session, 2) were scheduled for first study session and did not show up, or 3) did not respond to phone calls or emails of research staff.

### Procedures

The Indiana University and St. Vincent Indianapolis Hospital Human Subject Review Boards approved this study. In this prospective, observational study, participants completed three laboratory visits. The first visit occurred within 2 weeks post injury while visits 2 and 3 occurred approximately 1-month and 4-months post injury, respectively. During visit 1, participants reviewed and signed a written informed consent form approved by the IRB. To verify participants met inclusion/exclusion criteria, participants completed a health history questionnaire supplemented by interview. During each visit, participants completed the same questionnaires and quantitative sensory tests (QST). These assessments are described below. All participants were asked to refrain from pain-relief medication and consuming caffeine on the day of testing before their session.

#### Measures of pain modulatory function

Prior to each QST test, subjects were made familiar with each sensory test to be performed and were taught the 0–100 pain rating system. The tests of central sensitization (pressure pain hyperalgesia, temporal summation of pain) were performed first followed by the CPM test. A minimum of 10 min separated each central sensitization and CPM test.

#### Centra l sensitization measures

Several quantitative sensory tests in human experimental studies have been used to identify the presence of central sensitization including temporal summation of pain and generalized pressure hyperalgesia [20].

##### Pressure pain Hyperalgesia of the head/neck area

Pressure pain thresholds (PPTs) were tested on the following five sites of the head and neck areas, as has been conducted in prior research [9]: 1) middle of the forehead, 2) left temple, 3) parietal area (top of head), 4) posterior neck/C2, and 5) left trapezius. A digital, handheld, clinical grade pressure algometer (AlgoMed, Medoc Advanced Medical Systems, Durham, NC, USA) with a 1.0 cm^2^ probe was placed against the skin of one of the five sites and pressure was gradually increased at a slow constant rate of pressure (30kPA/s). The participant was instructed to verbally signal when (s) he first experienced pain caused by the pressure device at which time the algometer was removed. Two trials were performed at each site with twenty-second intervals between each trial. The PPTs at all sites were averaged for a single PPT score (PPT-Head) to be used in the data analysis [9].

##### Mechanical temporal summation (MTS)

Temporal summation is an indirect method of evaluating hyperexcitability of the central nervous system [20]. Mechanical temporal summation was tested on the back of the hand and on the forehead using the Von Frey filament (Touchtest Sensory Evaluator 6.65) calibrated to bend at 300 g of pressure. First, a single pinprick was applied with the von Frey filament to the body site. Participants rated the perceived pain intensity using a numeric rating scale (NRS) of 0 (no pain at all) to 100 (worst pain imaginable). Then, a series of 10 pinprick stimuli using the same monofilament was applied to the body site within an area of 1 cm^2^ and at a rate of 1 tap per second. Participants were asked to immediately rate the greatest pain intensity experienced during the 10 pinprick stimuli using the 0 to 100 NRS. The temporal summation value was calculated as the difference between the pain rating after the 10 stimuli and the first stimuli. This procedure was repeated twice at each body site with a 60-s rest interval between trials. The two trials at each site were averaged for a single MTS hand and MTS forehead score.

#### Conditioned pain modulation (CPM)

The most frequently used test of endogenous pain inhibition in humans is condition pain modulation (CPM). CPM refers to the reduction of pain produced by a test stimulus by a second noxious conditioning stimulus in a remote body site (i.e., “pain-inhibition-by-pain”) [21, 22]. For the CPM test, pressure pain thresholds (test stimulus) on the left arm were measured before and immediately following the submersion of the right hand in a cold water bath (conditioning stimulus). Seven minutes separated the pre PPT trials and the initiation of the conditioning stimulus, during which the participants sat quietly. This period of rest was included to prevent within-session adaptation, as prior work has shown complete recovery of primary afferent responsiveness after 10 min of no pain stimulation [23]. *Test Stimulus:* The test stimulus was PPTs administered on the left volar forearm. Using a digital, handheld, clinical grade pressure algometer the experimenter applied a slow constant rate of pressure (30kPA/s) to the left volar forearm. Participants were instructed to verbally indicate when the pressure sensation first became painful, at which the algometer was removed. Pressure pain threshold was defined as the amount of pressure in kilopascals (kPa) at which the participant first reported experiencing pain. Two trials were administered consecutively during each pre- and post-conditioning test. The posttest trials were administered immediately after participants removed their hand from the cold water bath (conditioning stimulus). These trials were averaged for a single pre- and post-test PPT score. *Conditioning stimulus*: Participants immersed their right hand up to the wrist in a cold-water bath (VersaCool 7, Thermo Scientific) maintained at 10 °C for 1 min. Cold pain was assessed every 15-s using the 0 to 100 NRS. The pain ratings were averaged across time for a single cold-water immersion pain score for each participant. *Calculation of CPM.* A percent change score was calculated for the test stimulus with the following formula: [(post PPT trial score – pre PPT trial score)/ pre PPT trial score]*100. A positive percent change score indicated an increase in PPTs following the conditioning stimulus and thus pain inhibition.

#### Psychological questionnaires

##### Center for Epidemiological Studies – Depression scale

The CES-D is a 20-item measure of symptoms of depression that has been shown to be reliable and valid in both general and clinical populations [24]. The score can range from 0 to 60, with higher scores indicating greater depression. A cut-off score of 16 or higher has been used as identifying individuals at risk for clinical depression [25].

##### Pain Catastrophizing scale (PCS)

The PCS assesses negative mental responses to anticipated or actual pain [26]. The PCS has 13 items that are scored on a Likert scale with three sub categories: rumination, magnification, and helplessness. Higher PCS scores are indicative of higher pain catastrophizing. Scores on the PCS have been associated with clinical and experimental pain measures. The highest possible score on the PCS is 52, with prior studies showing a cutoff range of more than 20–24 points to be related with clinical relevance [27, 28].

#### Classification of persistent posttraumatic headache

Participants were classified into a persistent PTH (PPTH) group and no persistent PTH (no-PPTH) group after all assessments were collected. This classification was made based on a standardized headache survey (described below) and in consultation with a neurologist.

##### Headache survey

A Headache Survey that has been used successfully in previous studies of post-traumatic headache was administered to all patients [5, 18]. The survey included questions about ongoing headache (frequency, intensity, duration, medication use, triggers, and other treatments), history of problems with headache preinjury, and characteristics of ongoing headache (headache symptoms). Participants rated the average pain intensity of their headaches during the past week using a 0 to 10 numeric rating scale (NRS), with 0 being no headaches at all and 10 being the worst pain possible.

##### Persistent PTH (PPTH) classification

Mild TBI participants were classified as having PPTH based on the International Classification of Headache Criteria 3rd edition (ICHD-3) for *persistent headache attributed to mild traumatic injury to the head*. Diagnostic criteria of PPTH attributed to mild head injury according to the ICHD-3 includes [3]:

(A) Headache fulfilling criteria C and D;
(B) Head injury fulfilling both of the following:
  1. associated with none of the following: a) loss of consciousness for > 30 min, b) Glasgow Coma Scale (GCS) score < 13, c) post-traumatic amnesia lasting > 24 h, d) altered level of awareness for > 24 h, e) imaging evidence of a traumatic head injury such as skull fracture, intracranial hemorrhage and/or brain contusion;
  2. associated with one or more of the following symptoms and/or signs: a) transient confusion, disorientation or impaired consciousness, b) loss of memory for events immediately before or after the head injury, c) two or more of the following symptoms suggestive of mild traumatic brain injury: i. nausea, ii. Vomiting, iii. Visual disturbances, iv. dizziness and/or vertigo, v. gait and/or postural imbalance, vi. impaired memory and/or concentration.
(C) Headache is reported to have developed within 7 days after one of the following: 1) the injury to the head, 2) regaining of consciousness following the injury to the head, 3) discontinuation of medication(s) impairing ability to sense or report headache following the injury to the head;
(D) Headache persists for > 3 months after its onset.
(E) Not better accounted for by another ICHD-3 diagnosis.

Participants who did not report headaches at 4 months post injury were classified into the no-PPTH group.

### Statistical analysis

Descriptive statistics were calculated for all the outcome variables. The Chi-square test was conducted to determine whether differences in sex existed between the PPTH and no-PPTH groups. Independent-t tests examined group differences between BMI and age. First, we sought to determine differences in the primary outcome measures in mild TBI patients who developed PPTH’s compared to mild TBI patients who did not develop PPTH’s at 1-week, 1-month, and 4 months post injury. Shapiro-Wilk’s test of normality indicated that the TS variables were not normally distributed. Thus, Mann-Whitney U tests were conducted to determine if TS at the hand and forehead differed between the PPTH and No-PPTH groups at each time point. We conducted 2(Group) × 3(Time) mixed model ANOVAs with repeated measures on time on CPM score, PPT-Head, PCS score, CES-D score, and HA-intensity score. Due to difficulties with the algometer for one participant visit, the no-PPTH group had data for one participant missing in the analysis for PPT-Head. Because the Chi-square test indicated that sex differed between groups, sex was considered as a covariate in each analysis. Tukey’s post hoc tests were used for significant main effects or interactions. The *p*-value for significance was set at *p* < 0.05.

Secondly, we used multiple logistic regression to identify baseline (1–2 week) predictors of the presence of PPTH at 4 months post injury (0 = no-PPTH group, 1 = PPTH group). Due to the large number of potential predictors, the initial choice of the independent variables for the model was based on the results of the Chi-square tests and ANOVAs discussed above. Because sex was a potential confounding variable, sex was added into the model first. Other primary outcome variables with *p* < 0.10 were retained in the final model. The goodness of fit of the final multivariate model was evaluated by Hosmer–Lemeshow (H–L) test, with *p* > 0.05 representing the acceptable result. The *p*-value for significance was set at *p* < 0.05.

## Results

### Participant characteristics

Forty-four participants completed the entire study. Two participants were removed from the data analysis for the following reasons: 1) one participant reported conflicting data on headaches at 4 months and 2) another participant reported headaches at 4 months, but the headaches were not worse than their headaches prior to the injury. Thus, 42 participants were included in the data analysis. Twenty-one participants met the criteria for PPTH and 21 participants were in the no-PPTH group. A power analysis using G-Power indicated that a total sample size of *n* = 42, with an alpha = .05 and power of .80 would allow detection of a moderate effect size (f = 0.27) for the within-between interaction of the mixed model ANOVA.

Means ± standard deviations and *p*-values for participant characteristics are presented in Table 1. The PPTH group contained significantly more females than the No-PPTH group, but no differences existed in age or BMI between groups. Reported causes of head injury included: falls, motor vehicle crashes, hit by object on head, hit by car/truck, hit head on wall, snow board accident, and bike accidents. Nineteen mild TBI participants reported loss of consciousness for under 30 min following head injury. Thirty-nine of 42 mild TBI participants reported headaches at visit 1, whereas 21 participants reported headaches at visit 3 (PPTH group). During visit 1, 36 participants reported taking medications for their headaches including triptans [1], NSAIDS [21], acetaminophen [17], opiates (6: short-term use only, as chronic use is an exclusion), and medicine that treats muscle spasms [4]. At 1-month post injury, 28 participants reported taking medications for their headaches including triptans [2], NSAIDS [22], acetaminophen [10], opiates (2, short-term use only, as chronic use is an exclusion), medicine that treats muscle spasms [2], and anti-anxiety [1] and antidepressant [1] medicine. At 4-months post injury, 20 participants reported taking medications for their headaches including triptans [2], NSAIDS [14], acetaminophen [6], medicine that treats muscle spasms [1], and anti-anxiety [2] and antidepressant [1] medicine. All participants reported taking medicine for abortive purposes only (i.e. only using medications when a headache was present). However, these medications were not taken on the day of testing, before the session took place.

**Table 1:** Participant Characteristics

	***PPTH Group (n = 21***	***No-PPTH (n = 21)***	
Variable	Mean ± SD	Mean ± SD	*p*-value
Age, years	29.5 ± 9.4	32.9 ± 9.8	0.253
Sex, % female	66.7%	33.3%	0.013*
Body Mass Index	28.7 ± 9.2	26.8 ± 4.9	0.414
Education			
Some HS	5.3%	9.5%	
HS degree	47.4%	33.3%	
2 year College degree	15.8%	9.0%	
4 year College degree	15.8%	28.6%	
Masters or above	15.8%	9.5%	
Race			
African American	38.1%	14.3%	
Asian/Pacific Islander	0.0%	4.8%	
Caucasian	52.4%	66.7%	
Hispanic	4.8%	14.3%	
Other	4.8%	0.0%	
Frequency of headaches at 1–2 weeks post injury (since injury)			
No headaches since injury	0.0%	14.7%	
About once a week	4.8%	19.0%	
Several times a week	47.6%	33.3%	
Daily	42.9%	33.3%	
Constant	4.8%	0.0%	
Frequency of headaches at 1-month post injury (past month)			
No headaches since 1-week visit	0.0%	33.3%	
About once a month	14.3%	19.0%	
About once a week	33.3%	19.0%	
Several times a week	23.8%	23.8%	
Daily	23.8%	4.8%	
Constant	4.8%	0.0%	
Frequency of headaches at 4-months post injury (past 3 months)			
No headaches since 1-month visit	0.0%	57.1%	
Less than once a month	0.0%	42.9%	
At least once a month	14.3%	0.0%	
About once a week	52.4%	0.0%	
Several times a week	28.6%	0.0%	
Daily	4.8%	0.0%	
Constant	0.0%	0.0%	

*HS* high school, *PPTH* Persistent posttraumatic headache;^*^significant *p*-value

### Differences in primary outcome variables across time in the PPTH and no-PPTH groups

Table 2 presents the means and standard errors (SE) of all primary outcome variables across time for the PPTH group and no-PPTH group. Data presented in the text represent mean ± SE.

**Table 2:** Means and SE’s of primary outcome variables across time for the PPTH and no-PPTH group

Variable	1–2 weeks post injury	1-month post injury	4 months post-injury
**Headache Variables**			
HA-Intensity (0–10 scale)**			
No-PPTH	4.2 ± 0.5	2.2 ± 0.5	0.0 ± 0.0
PPTH	7.0 ± 0.5	6.1 ± 0.5	6.3 ± 0.4
**Measures of Pain Modulatory Function**			
*TS Hand*			
No-PPTH	15.6 ± 3.4	15.8 ± 3.8	12.4 ± 4.1
PPTH	10.0 ± 3.4	13.8 ± 3.8	13.3 ± 4.1
*TS Forehead*			
No-PPTH	16.1 ± 3.8	18.5 ± 3.6	16.6 ± 4.3
PPTH	14.7 ± 3.8	15.5 ± 3.6	17.7 ± 4.3
*PPT-Head*			
No-PPTH	236.2 ± 26.1	246.4 ± 25.5	242.1 ± 23.8
PPTH	199.7 ± 25.4	232.3 ± 24.8	226.2 ± 23.1
CPM Score, %*			
No-PPTH	33.6 ± 6.8	28.2 ± 4.4	26.7 ± 6.5
PPTH	5.6 ± 6.8	11.1 ± 4.4	18.9 ± 6.5
**Psychological Variables**			
Pain Catastrophizing Scale Score*			
No-PPTH	15.3 ± 2.3	9.9 ± 2.4	7.0 ± 2.3
PPTH	24.6 ± 2.3	20.1 ± 2.4	17.7 ± 2.3
CES-Depression scores*			
No-PPTH	19.0 ± 2.7	11.7 ± 2.0	9.5 ± 2.4
PPTH	24.1 ± 2.7	18.9 ± 2.0	19.2 ± 2.4

^**^significant Time by Group interaction, ^*^significant Group main effect; *SE* Standard error, *PPTH* persistent posttraumatic headache, *HA* headache, *TS* temporal summation, *PPT* Pressure pain threshold, *PCS* Pain Catastrophizing Scale, *CPM* conditioned pain modulation

#### Headache pain intensity

The mixed model ANOVA on headache pain intensity revealed a significant effect of time (*p* < .001) and group (*p* < .001), which was superseded by a significant time by group interaction (*p* < .001). The follow-up tests indicated that headache pain intensity significantly decreased from 1 to 2 weeks to 1 month to 4 months post injury in the no-PPTH group. No changes across time were found for the PPTH group. Also, the PPTH group had significantly greater headache pain at all time points compared to the no-PPTH group.

#### Measures of pain modulatory function

The non-parametric tests showed that TS of the hand and forehead did not significantly change across time or differ between groups at any time point (p’s > .43). The ANOVA conducted on PPT-head also did not demonstrate any significant effects (main effect of time *p* = .645; main effect of group *p* = .503; interaction *p* = .677). For CPM score, the ANOVA revealed a significant main effect of group (*p* = .001), with the no-PPTH group (M = 29.5 ± 3.6) exhibiting greater pain inhibition on the CPM test compared to the PPTH group (M = 11.9 ± 3.6). The main effect of time (*p* = .824) and interaction were not significant (*p* = .232).

#### Psychological variables

For pain catastrophizing, the mixed model ANOVA showed a significant effect of group (*p* = .001). The PPTH group (M = 20.8 ± 1.9) exhibited higher pain catastrophizing compared to the no-PPTH group (10.7 ± 1.9), regardless of time point. The main effect of time (*p* < .001) was also significant, with pain catastrophizing significantly greater at 1-week post injury (19.9 ± 1.6) compared to 1-month (14.9 ± 1.7) and 4 months post injury (12.3 ± 1.6). The interaction was not significant (*p* = .900). The ANOVA conducted on CES-Depression scores revealed a main effect of group (*p* = .016). The PPTH group (M = 20.7 ± 2.1) reported higher depression scores compared to the no-PPTH group (M = 13.4 ± 2.1). The main effect of time (*p* < .001) was also significant, with depression scores significantly greater at 1-week post injury (21.6 ± 1.9) compared to 1-month (15.3 ± 1.4) and 4 months post injury (14.4 ± 1.7). The interaction was not significant (*p* = .271).

### Predicting persistent posttraumatic headache

The final model for the multiple logistic regression included sex, headache pain intensity at 1-week, and CPM score at 1-week as independent variables. The overall model was statistically significant, *p* < .001. The inferential goodness-of-fit test was the Hosmer-Lemeshow test that yielded an insignificant result (*p* = .340), indicating that the model fit the data well. The results for the individual predictors are provided in Table 3. Headache pain intensity and CPM score were significant predictors, with greater headache pain intensity and lower CPM score predicting a higher likelihood of having PPTH at 4 months post-injury.

**Table 3:** Summary of multiple logistic regression results for prediction of PPTH at 4-months post-mild TBI

Variables	*B*	S.E.	Wald	*P* -value	Exp(B)	95% C.I.	
					(Odds Ratio)	Lower	Upper
1. Sex	−.962	.884	1.18	.277	.382	.068	2.16
2. HA-intensity 1-week	.684	.273	6.21	.013*	1.97	1.16	3.37
3. CPM score 1-week	.037	.016	5.21	.022*	.963	.933	.995

^*^statistically significant; *PPTH* Persistent posttraumatic headache, *C.I.* Confidence Interval, *HA* headache, *CPM* conditioned pain modulation.

## Discussion

The current pilot study is the first to prospectively evaluate whether endogenous pain modulatory function in the early stages following mild TBI predicts the development of persistent PTH’s. Several key findings emerged from this study. First, we found that persistent PTH is characterized by dysfunctional alterations in endogenous pain modulatory function in the early stages following mild TBI. Second, deficient endogenous pain inhibitory capacity at 1-week post injury predicted the presence of persistent PTH’s at 4 months post injury. Third, mild TBI patients with persistent PTH showed elevated psychological distress across the 4-months post injury compared to mild TBI patients not developing persistent PTH.

### Role of endogenous pain modulatory function in persistent PTH’s

Preclinical and human studies suggest that mild TBI is characterized by diminished pain inhibitory capacity in the early stages following injury [12–14]. For example, a recent rodent study indicated that mild TBI may cause profound disruption of descending noxious inhibitory controls (DNIC). DNIC is the corresponding test in animals to CPM in humans [22]. The mechanism for diminished pain inhibition following mild TBI is unknown; however, it could be associated with TBI-induced damage to the ascending spinothalamic/ thalamocortical tracts in the brain [29]. Furthermore, prior research suggests that brain regions involved in the major descending pain inhibitory tracts, such as the brain stem, are particularly susceptible to damage following TBI [30]. This TBI-induced damage may diminish the descending inhibitory control that is elicited by ascending nociceptive information [31]. Importantly, our findings provide novel evidence that mild TBI patients developing persistent PTH exhibit significantly diminished pain inhibitory capacity following injury compared to those who do not develop persistent PTH. Furthermore, we showed that this diminished endogenous pain inhibitory control within 2 weeks post-injury places mild TBI patients at an increased risk for developing persistent PTH’s at 4 months post injury. These results are in line with Defrin et al., who demonstrated cross-sectionally that mild TBI patients at least 1 year post-injury with chronic PTH’s had diminished pain inhibitory capacity on the CPM test compared to mild TBI patients without headaches and mild TBI free individuals [15]. Overall, our findings add to the accumulating body of research suggesting that individuals with impaired engagement of descending pain inhibitory pathways are at an increased risk for developing chronic pain [11, 32].

We also hypothesized that sensitization of the head/neck area, as measured by quantitative sensory tests, at 1–2 weeks post injury would play a role in persistent PTH’s. Prior research indicates that central sensitization is characteristic of many chronic pain conditions [9, 33, 34] and may be a risk factor for the development of intense and chronic pain [35]. Furthermore, our prior study had demonstrated greater pressure pain hyperalgesia of the head in the mild TBI patients compared to controls at 1–2 weeks post injury, with greater pressure pain hyperalgesia associated with headache pain within the mild TBI group [13]. Rodent studies have also revealed sustained sensitization to mechanical stimuli in the head area and distal sites in mice following mild TBI [12, 36]. However, our hypothesis was not supported. Pressure pain sensitivity and temporal summation of pain did not predict persistent PTH’s and were not significantly facilitated in the PPTH group compared to the no-PPTH group. These results are in contrast to a cross-sectional human study on primary headaches demonstrating the presence of sensitization of the head as measured by both PPT’s and temporal summation of pain in patients with chronic migraine and tension-type headaches compared to controls [9]. It is possible that longer maintenance of headaches facilitate greater central sensitization, rather than central sensitization being a contributing causal factor to persistent PTH. Perhaps with a longer maintenance of PTH, the sensitization measures would significantly differ between those with and without PTH’s in the chronic phase. However, larger studies with longer follow-up are needed to test this hypothesis.

### Psychological factors as predictors of posttraumatic headache

Mild TBI is often accompanied by psychological distress [37], including high levels anxiety, depression, and pain catastrophizing [13]. In general, a substantial amount of evidence supports the importance of these psychological factors in shaping pain-related experiences. However, no prior research has longitudinally evaluated pain catastrophizing and posttraumatic headaches following mild TBI. We provided evidence that mild TBI patients who develop persistent PTH’s report higher pain catastrophizing and depression at 1-week, 1-month, and 4-months post-injury compared to those who do not develop persistent PTH’s. These findings are in accordance with prior research showing an increased co-occurrence of depression and PTH’s over time [38]. Additionally, Chaput and colleagues demonstrated cross-sectionally that pain catastrophizing was associated with adverse mild TBI outcomes at 8 weeks post mild TBI, including headache pain [17]. Notably, while depression and pain catastrophizing were elevated in the PPTH group, our findings indicated that these psychological processes at 1–2 weeks post injury did not increase the risk for the development of persistent PTH’s. One alternative explanation is that perhaps these psychological factors contribute to the intensity of headaches experienced vs. the maintenance of headaches over time. Indeed, non-TBI injury-related research has shown that pain catastrophizing at baseline predicts pain severity over 12-months following non-catastrophic injuries [39]. A second possibility is that PTH’s contribute to increased depression and pain catastrophizing versus these psychological factors increasing the risk for persistent PTH’s. In the current study, average depression scores in the PPTH group at each time point were higher than the recommended cut-off score for identifying risk of clinical depression. Importantly, past research has independently linked depression, headaches, and mild TBI to psychosocial impairment [40] and negative outcomes, such as suicidal ideation and behavior [41–43]. Thus, future research should elucidate how these three variables interactively influence the risk of suicidal behavior and ideation. Furthermore, future research should consider known personality (i.e., affective temperament [43]) and environmental/behavioral (e.g., childhood maltreatment [43]) risk-factors in identifying mild TBI patients at highest risk for these negative psychological outcomes (i.e., depression, suicide).

### Other factors as potential predictors of posttraumatic headache

While not the primary focus of the current study, we also evaluated other potential variables as predictors of persistent PTH. Notably, headache pain intensity at 1–2 weeks post injury increased the risk for persistent PTH, such that every 1-point increase in headache pain nearly doubled the risk for being in the PPTH group. Additionally, the PPTH group reported significantly greater headache pain in the early phase following injury compared to the no-PPTH group. However, the no-PPTH group averaged over a 4 on a 0–10 NRS scale at 1–2 weeks, indicating moderate headache pain still existed in the early phase post injury in those not developing chronic headaches. Other research has also shown the importance of acute pain in the development of chronic pain. For example, a large prospective observational study of PTH following mild and moderate TBI found that having a headache during the emergency department visit for the head injury (all participants recruited from the ED) was a risk factor for persistent PTH [44]. Research investigating the chronification of pain following fracture injuries also demonstrates that acute pain in the first weeks following injury is an important predictor of chronic pain [45–47].

We also found a greater percentage of females in the PPTH group compared to the no-PPTH group. However, sex was not a significant predictor in the regression model. Research examining sex as a risk factor for persistent PTH has shown mixed results. A recent preclinical study found that traumatic head injury leads to an enhanced and prolonged cephalic hyperalgesic response in female compared to male rats [48]. Along these lines, some clinical research indicates that more women experience post-concussion symptoms (e.g., headaches) and complications following mild TBI compared to men [49, 50]. Additionally, Yilmaz and colleagues identified being a female as a significant risk factor for the presence of PTH 3-months after mild and moderate TBI [44]. In contrast, Mollayeva et al. found no sex differences in the frequency of head/neck and bodily pain for several months following mild TBI [51]. Given the contrasting results, further research is needed to elucidate the contribution of sex to persistent PTH susceptibility.

### Limitations

A few limitations of this study should be noted. First, all participants were recruited from the Emergency Department and we excluded patients with fracture or polytrauma. Thus, we do not know whether the current study’s results would generalize to mild TBI patients not seen at the ED or those with multiple injuries at the time of head injury. Secondly, we only followed participants for 4 months. Predictors of PTH’s at a longer duration post injury (i.e., 12 months) could be different from those found in this study. Third, at the four-month assessment, headaches were assessed retrospectively with an interview versus using a headache diary. Additionally, while we consulted with a neurologist on PTH classification, the neurologist did not personally conduct the headache interviews. Finally, no single defining, clinical characteristic exists for post-traumatic headache [52]. Prospective studies of post-traumatic headaches indicate migraine followed by tension-type headaches are the most common headache types in mild TBI patients [5]. Neuroimaging and electroencephalography studies suggest different and overlapping pathophysiological mechanisms underlying migraine and PTH [53]. As in the current study, very few PTH studies investigate the possibility of differential risk factors for PTH’s of various clinical presentations (i.e., migraine vs. tension-type, vs. mixed). Importantly, prior research has shown that central sensitization and deficient pain inhibition exists in migraine and tension-type headache patients, suggesting a common role for abnormal pain modulation in the chronification of different types of headache [9, 20]. However, future studies with larger sample sizes should still explore the possibility of both common and different underlying neurophysiological mechanisms underlying persistent PTH of different phenotypes.

## Conclusions

The transition from acute to persistent PTH following mild TBI likely involves a complex interaction of neurobiological processes. Consequently, the medical management of the condition is unsatisfactory with current treatments often ineffective [54]. Indeed, no specific pharmacological therapy currently exists for persistent PTH [See Guglielmette et al. for a Review of PPTH treatments: [40]]. Clinicians typically prescribe headache medications used for primary headache disorders for PTH, but studies show that few PTH patients derive relief from these pharmaceutical preparations [54, 55]. As such, evidenced-based approaches to persistent PTH prevention and management are greatly needed in which mechanisms and other factors contributing to headache pain are identified to guide treatment. Importantly, the current study provides the first longitudinal human evidence for the contribution of deficient endogenous pain inhibition in the transition from acute to persistent PTH. Future prospective and multidisciplinary studies with increased numbers of subjects are needed to confirm the current study’s results. However, once potential treatment targets are identified, such as deficient pain inhibition, a second step will be to test the efficacy of mechanistic based treatments (e.g., pharmacological agents aimed at increasing inhibitory tone) to prevent or manage persistent PTHs. Notably, other studies have shown that identifying the dysfunctional pain modulatory state can be instrumental in the choice of drug for pain alleviation [11].

## Data Availability

The datasets used and/or analysed during the current study are available from the corresponding author on reasonable request.

## Abbreviations

PTH: Posttraumatic headache
TBI: Traumatic brain injury
PPTH: Persistent posttraumatic headache
CPM: Conditioned pain modulation
ED: Emergency deparment
IRB: Institutional review board
QST: Quantitative sensory testing
PPT: Pressure pain threshold
MTS: Mechanical temporal summation
NRS: Numeric rating scale
CES-D: Center for Epidemiological Studies Depression
PCS: Pain Catastrophizing Scale
ICHD-3: International Classification of Headache Criteria 3rd edition
BMI: Body mass index
SE: Standard error
DNIC: Descending noxious inhibitory controls
